# Investigating the effectiveness of tele-counseling for the mental health of staff in hospitals and COVID-19 clinics: a clinical control trial

**DOI:** 10.47626/2237-6089-2020-0176

**Published:** 2021-11-09

**Authors:** Masumeh Ghazanfarpour, Farzane Ashrafinia, Shahrzad Zolala, Atefeh Ahmadi, Yunes Jahani, Ali Hosseininasab

**Affiliations:** 1 Student Research Committee Razi Faculty of Nursing and Midwifery Kerman University of Medical Sciences Kerman Iran Student Research Committee, Razi Faculty of Nursing and Midwifery, Kerman University of Medical Sciences, Kerman, Iran.; 2 Department of Midwifery Razi Faculty of Nursing and Midwifery Kerman University of Medical Sciences Kerman Iran Nursing Research Center, Department of Midwifery, Razi Faculty of Nursing and Midwifery, Kerman University of Medical Sciences, Kerman, Iran.; 3 Department of Counselling in Midwifery Razi Faculty of Nursing and Midwifery Kerman University of Medical Sciences Kerman Iran Nursing Research Center, Department of Counselling in Midwifery, Razi Faculty of Nursing and Midwifery, Kerman University of Medical Sciences, Kerman, Iran.; 4 Modeling in Health Research Center Institute for Future Studies in Health Kerman University of Medical Sciences Kerman Iran Modeling in Health Research Center, Institute for Future Studies in Health, Kerman University of Medical Sciences, Kerman, Iran.; 5 Infectious and Tropical Research Center Kerman University of Medical Sciences Kerman Iran Infectious and Tropical Research Center, Kerman University of Medical Sciences, Kerman, Iran.

**Keywords:** Coronavirus, tele-counseling, staff, hospitals, clinics, anxiety, depression, health anxiety

## Abstract

**Objective:**

To investigate the effectiveness of tele-counseling for the mental health of staff working in hospitals and reference clinics during the COVID-19 outbreak.

**Methods:**

In the first stage of the study, using a convenience sampling strategy, 313 staff members working at Iran’s hospitals and COVID-19 clinics answered a Hospital Anxiety and Depression Scale and the Short Health Anxiety Inventory online. In a second stage, 95 staff members who were willing to participate in the intervention were randomly assigned to the intervention (n = 51) or control (n = 44) groups. The intervention consisted of seven intensive tele-counseling sessions.

**Results:**

In the first stage, the percentages of anxiety and depression related to coronavirus were 79.2% and 82.1% and the mean health anxiety score was 17.42. In the intervention phase, anxiety related to coronavirus and to perceived risk of illness (likelihood of illness) were significantly lower in the intervention group in comparison with the control group (p = 0.001). Depression related to coronavirus and anxiety related to the negative consequences of infection were non-significantly reduced in the intervention group compared to the control group (p = 0.08 and 0.12; respectively).

**Conclusion:**

Continuous monitoring of the negative psychological impacts on medical staff of outbreaks as well as implementation of appropriate interventions to respond to them should be emphasized in order to improve staff mental health.

Clinical trial registration: Iranian Registry of Clinical Trials, IRCT20170611034452N11.

## Introduction

### Scientific background and explanation of rationale

Occupational health and mental health have significant impacts on each other. During epidemics, health care providers are continually exposed to the factors associated with the risk of developing mental disorders such as stress, anxiety, and depression. Being infected or fear of infection have been significantly associated with absenteeism, leaving the workplace, negative attitudes, and decreased efficiency and performance of medical staff.^1^ Infections and occupational injuries can lead to more severe forms of distress response in epidemic conditions.^2,3^

In pandemics, especially in the case of newly emerging epidemics in which treatment and safety protocols are yet to be properly investigated, a large number of patients are hospitalized and the medical team is subjected to a heavy burden imposed by workload, anxiety, and fear related to the concern of being infected.^4^ Unknown infections with unknown transmission routes, rapid global prevalence, and relatively high mortality can affect health care staff more than members of other organizations. These conditions may have a potentially deleterious impact on physical and mental health, ability to manage crises, and performance in patient care delivery.^5^ In this scenario, mood disorders, insomnia, perceived negative emotions, and post-traumatic stress disorder are among the problems that can affect the quality of life of staff.^6^

The novel beta-coronavirus, associated with a series of respiratory system symptoms and later named SARS-CoV-2, emerged in Wuhan, China, in December 2019 and spread rapidly to other countries and, in spite of interventions, this virus continues to infect populations all over the world.^7,8^ The increased number of confirmed or suspected cases, heavy workload, lack of personal protective equipment, excessive media coverage, lack of specific medications, and feelings of insufficient support all increase the psychological burden on medical staff.^9^ Moreover, feelings of vulnerability, loss of control, concerns about personal health and transmitting the virus to family members and others, occupational change and fear of isolation, rapid human-to-human transmission of the virus, and the high mortality rate of the infection all increase staff’s awareness of the risk they are taking.^10^ Furthermore, factors such as age, gender, marital status, and the type of hospital and service department are associated with the severity of fear, anxiety, sadness, anger, and sleep disorder symptoms in hospital staff.^11^

Lack of social support, awareness, adaptability, and calming strategies increases the emergence of negative psychological outcomes.^12^ The feeling of occupational pressure and stress in the coronavirus crisis necessitates intervention to improve mental health by teaching management and coping strategies.^13^ Advising this group to maintain safe interactions and providing social support will help reduce the psychological burden of occupational exposure.^14^

To address the psychological needs of medical professions, an intervention program involving identification of stressors and promotion of emotion regulation and problem solving skills can reduce anxiety.^15^ For instance, mindfulness-based interventions try to improve mental health by reducing the overall stress a person experiences.^16^ Furthermore, these interventions increase awareness, enhance coping strategies, alleviate the negative effects of epidemics,^17^ and moderate perceived stress and depression.^18^ Psychological methods for monitoring thoughts in the face of critical situations can relieve symptoms of mood disorders, by creating new beliefs to curb cognition errors and behavioral mistakes.^19^ During the coronavirus epidemic, when social distancing is unavoidable, online systems for providing mental health care services are extremely important.^20^

Burdened by heavy workloads and exhaustion as well as anxiety and depression, the medical staff in Iran suffer from high rates of infection, mortality, and morbidity from COVID-19 infection.^21^ To date, health care providers in many countries have not received any training to maintain their mental health in optimum condition.^22^ So far, no studies have been published on providing staff with tele-counseling for coping with the distress and tension of the COVID-19 pandemic in order to reduce its negative consequences.

### Specific objectives

The aim of this study was to investigate the effectiveness of tele-counseling for improving the mental health of staff in hospitals and COVID-19 clinics in Iran.

## Methods

### Trial design

This study is a randomized controlled trial with one intervention group and one control group, conducted from March 10 to March 30, 2020. All health care providers in private and state hospitals and COVID-19 clinics in the southern half of Iran were invited to participate in this study using a convenience sampling method.

The invitation to participate was distributed in several ways. Firstly, a letter was sent from the vice president of Kerman University of Medical Sciences to the vice chancellors of health and curative affairs. Requesting them to encourage their staff to fill out the online questionnaires. Secondly, an invitation letter containing the link was shared with the research team’s professional and social networks and personal connections and snowball sampling was employed to invite health care providers as well.

In the second phase, medical staff who indicated their willingness to participate in the intervention on the questionnaire in the previous stage were contacted and invited to participate. Then they were randomly allocated by the study’s statistics consultant using a random number table (allocation ratio: 1/1) to the intervention group (n = 51) or the control group (n = 44). Those assessing outcomes were blinded to the study.

### Eligibility criteria for participants and settings and locations

No limitations on age, specialty, place of work, or years of work experience were used to restrict participation in this study. All of the staff working in hospitals and COVID-19 clinics were eligible to participate in the first and second stages. The exclusion criteria were as follows: (a) history of severe physical and psychiatric disorder preventing the participant from active participation in the sessions, (b) participation in other psychological interventions that could influence anxiety, depression, and health anxiety, and (c) absence from more than two sessions.

### Outcome measurements

The primary outcome of this study was the frequency of anxiety and depression related to coronavirus and health anxiety (anxiety in two domains caused by the perceived risk of illness as well as associated negative consequences of infection). The secondary outcome was the effect of tele-counseling on the levels of depression and anxiety related to the coronavirus pandemic as well as two domains of heath anxiety.

### Ethical considerations

The study was approved by the medical ethics committee at Kerman University of Medical Sciences (IR.KMU.REC.1398.737) and registered with the Iranian Registry of Clinical Trials (IRCT20170611034452N11). The study was also conducted in accordance with the Declaration of Helsinki. Participants were told that they were able to withdraw from participation at any point of time, no reason required. All data were recorded in a manner that protected the anonymity of the participants. The online questionnaire began with a general description of the study and the questionnaire included a button with which the participant could indicate that they provided their informed consent. The investigator’s contact information was given at the end of the online questionnaire, in case participants had any questions about the study. In addition, staff who were willing to take part in the second stage informed the researcher. Control group members could request to participate in similar sessions to those provided to the intervention group after the index intervention had been completed.

### Measurements

#### Demographics

The information on the sociodemographic data collection form was selected based on the relevant literature as well as the researchers’ experience. The findings are presented in Table 1.

**Table 1:** Demographic and clinical characteristics of subjects in the two study groups

Variable	Intervention	Control	p-value
Age			
< 30	37 (72.5.9)	28 (63.6)	0.239
≥ 30	14 (27.5)	16 (36.4)	
Gender			
Male	44 (86.3)	36 (81.8)	0.553
Female	7 (13.7)	8 (18.2)	
Working in coronavirus patients’ wards			
No	34 (66.7)	35 (79.5)	0.160
Yes	17 (33.3)	9 (20.5)	
Being in contact with coronavirus patients			
No	37 (72.5)	31 (70.5)	0.821
Yes	14 (27.5)	13 (29.5)	
Being in contact with patients suspected of having coronavirus			
No	19 (37.3)	16 (36.4)	0.928
Yes	32 (62.7)	28 (63.6)	
Having any symptom of coronavirus at present			
No	47 (92.2)	35 (79.5)	0.075
Yes	4 (7.8)	9 (20.5)	
Living with an elderly person (> 65 y)			
No	43 (84.3)	38 (86.4)	0.779
Yes	8 (15.7)	6 (13.6)	
Suffering from a chronic disease			
No	51 (100)	42 (95.5)	0.124
Yes	0 (0.0)	2 (4.5)	
Suffering from a documented psychiatric disorder			
No	45 (88.2)	40 (90.9)	0.672
Yes	6 (11.8)	4 (9.1)	

Data presented as n (%).

#### The Hospital Anxiety and Depression Scale (HADS)

The HADS was developed by Zigmond and Snaith in 1983 and is a self-report tool used to measure both anxiety and depression. The tool comprises 14 items on two subscales, seven related to anxiety (HADS-A) and seven related to depression (HADS-D).^22^ Each item is answered on a 4-point Likert scale (scores ranging from 0-3) and the total score of each subscale ranges from 0 to 21. The cutoff score for both anxiety and depression is 7.^23^ The tool has been validated in different languages and cultures. The Persian version of HADS, translated by Montazeri et al., was used in this study. Montazeri et al. reported that the HADS scale significantly discriminated between anxiety and depression. The results for convergent validity showed a significant negative correlation between the Cancer Quality of Life Questionnaire (EORTC QLQ-C30) and the HADS. HADS has been validated in different fields such as in cancer,^23^ infertility,^24^ and epilepsy^25^ in the Iranian population. It is sensitive to mood changes during the course of therapy in response to psychotherapeutic and psychopharmacological intervention.^26^ The health care staff were requested to answer all the questions specifically in relation to “the effects of coronavirus”, in order to assess anxiety and depression specific to this epidemic.

#### Short Health Anxiety Inventory (SHAI)

The SHAI is an 18-item self-report tool developed by Salkovskis et al., in 2002 to assess health anxiety along a four-point Likert scale (0 to 3). Total scores range from 0 to 54; with higher scores indicating greater symptomology. The first 14 items are related to mental concern and frequent encounters with health issues (likelihood of illness domain). The remaining 4 items (the negative consequences domain) deal with people’s attitudes towards how awful it would be if they developed a serious illness.^27^ Nargesi et al. assessed the validity of the Persian version of the SHAI in a sample of university students, reporting a Cronbach’s alpha of 75%.^28^

## Sample size calculation

In the first stage of the study, the sample size calculation formula for cross-sectional studies was used to calculate the minimum sample size necessary for measuring the prevalence of anxiety, depression, and health anxiety among the staff of hospitals and COVID-19 clinics. Considering α = 0.05; *d* = 0.06 and *P* = 0.50^29^; the sample size was calculated at 267 people. Accounting for a 15% probability of drop-outs and incomplete questionnaires, the final sample size was calculated as 308 people.


$$\begin{array}{c} n=\frac{\left(z_{1-\frac{\alpha }{2}}^{2}\times p\times (1-p)\right)}{d^{2}}\\ \alpha =0.05,z_{1-\frac{\alpha }{2}}=1.96 \end{array}$$

In the second stage, to calculate the minimum sample size for the intervention group (those taking part in tele-counseling sessions), the two-point comparison formula was used based on a similar study conducted in China in 2019.^30^ The sample size calculated for each group was 8 people. In order to increase the study’s power, accounting for possible drop-outs during the intervention by performing parametric probability distribution tests, at least 30 samples were required for each group.


$$n_{1}=n_{2}=\frac{{\left(z_{\alpha /2}+z_{\beta }\right)}^{2}\left(s_{1}^{2}+s_{2}^{2}\right)}{{\left({\bar{x}}_{1}-{\bar{x}}_{2}\right)}^{2}}$$

## Psychological intervention

### Aim of counseling

To manage anxiety and depression related to the COVID-19 outbreak and the resulting health anxiety of medical workers, the intervention implemented in this study was designed to provide information regarding workers’ safety; to be supportive and mindfulness-based; to clarify workers’ cognitive errors regarding the epidemic; to facilitate behavioral modification; and to improve workers’ mental health level.

### Intervention design

The psychological intervention implemented in this study is in line with recommendations made by Zhang et al. for responding to the COVID-19 epidemic.^31^ The content of the seven sessions was chosen according to standard cognitive-behavioral and mindfulness-based techniques.

### Counseling method

Medical workers who were allocated to the intervention group were divided into 21 WhatsApp groups. Counseling was implemented through voice or video calls, text chats, and video clips shared on WhatsApp, in seven sessions on seven consecutive days Depending on the number of questions and participants’ experience, the duration of audio and video exchange in each session varied from a minimum of 45 minutes up to 90 minutes. Control group members were able to request to participate in similar sessions after the index intervention had been completed.

### Counselors

The intervention was delivered by 21 trained counselors who were “Counseling in Midwifery” master’s students trained by the lead researcher, who is an associate professor in guidance and counseling, based on the study protocol.

### Study protocol

**Session 1:** Information regarding safety of medical staff and implementation of a healthy lifestyle in the recent outbreak via a psychoeducational approach; dysfunctional beliefs related to coronavirus-induced stress; relaxation techniques; introduction to mindfulness; “conscious eating” technique; and homework.

**Session 2:** Defining mindfulness and its benefits in the recent outbreak; mindful breathing techniques and meditation; personal borders and boundaries; systematic desensitization for anxiety starting from this session and continued in all subsequent sessions; and homework.

**Session 3:** Information regarding consequences of stress and anxiety via psychoeducational approach; cognitive errors of medical staff in the recent outbreak; overcoming occupational fatigue during epidemic workload; aerobic exercises to increase breathing capacity (helpful if infected in the future); and homework.

**Session 4:** Defining the automatic mind pilot and coping skills; meditation; mindful breathing; and homework.

**Session 5:** Re-discussing cognitive errors about worries and fears in epidemics; improving mindful intimacy and love; meditation; and homework.

**Session 6:** Discussion about accepting conditions and commitment in the recent outbreak; organizational mindfulness; body scan; and homework

**Session 7:** Talking about (pleasant and unpleasant) life events; related emotions and point of view toward them; self-care; describing how life is like the game of snakes and ladders; mountain meditation; reviewing and summarizing.

## Statistical analysis

Descriptive statistics were reported as frequency, percentage, and mean ± SD. The chi-square test was used to compare demographic variables between intervention group and control group, in view of their homogeneity. The paired t-test was used to compare anxiety and depression scores for each group measured in the initial screening phase with the same parameters at the end of the study. The independent t-test was used to compare the anxiety and depression changes (before-after) between the intervention and control groups. Data were analyzed using the Statistical Package for the Social Sciences (SPSS) version 22; and the significance level adopted was 0.05.

## Results

This study is a randomized controlled trial with one intervention group and one control group conducted from March 10 to March 30, 2020. In the first stage, 313 staff members filled out the questionnaires in an online survey. Following Montazeri et al.,^23^ a score of 7 was adopted as the cutoff score for both anxiety and depression. The percentages of anxiety and depression related to coronavirus among 313 staff in the first stage were 79.2% and 82.1%; respectively. The mean health anxiety score was 17.42 and mean scores for the two subscales, anxiety of likelihood of illness and its negative consequences, were 13.7 and 3.45, respectively.

**Figure 1:**
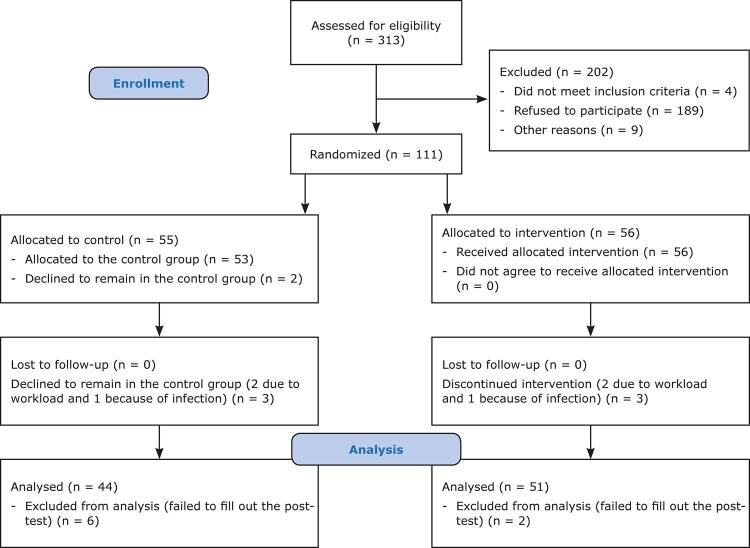
Flow diagram illustrating sample selection.

For the second stage, 202 phase one participants were excluded because they did not meet eligibility criteria for inclusion (n = 4) or refused to participate in the intervention (n = 189), or for other reasons (n = 9). 111 staff were randomly divided into the intervention (n = 51) and control (n = 44) groups (Figure 1). Table 1 shows demographic data, which were homogenous at baseline in two groups. Table 2 shows the results for comparisons of depression and anxiety related to coronavirus, anxiety of likelihood of illness, and anxiety of its negative consequences within and between the control and intervention groups.

**Table 2:** Comparisons within and between control and intervention groups of depression and anxiety related to coronavirus, anxiety of likelihood of illness, and anxiety of negative consequences

Variable	Before Mean ± SD	After Mean ± SD	p* (effect size; mean difference; 95%CI)	p^†^(effect size; mean difference; 95%CI)	Difference (before – after) Mean ± SD	p^‡^(effect size; mean difference; 95%CI)
Anxiety related to coronavirus						
Intervention	10.17±2.29	8.58±1.99	0.06 (1.89; 0.91; -0.04-1.84)	< 0.001 (5.06; 1.58; 0.95-2.21)	1.58±2.23	0.001 (3.41; 1.52; 0.63-2.4)
Control	9.27± 2.33	9.2±2.22		0.82 (0.21; 0.06; -0.56-0.69)	0.06±2.07	
Depression related to coronavirus						
Intervention	10.27±2.08	9.05±2.07	0.36 (0.92; 0.41; -0.47-1.29)	0.002 (3.24; 1.21; 0.46-1.96)	1.21±2.67	0.08 (1.76; 0.87; -0.11-1.87)
Control	9.86±2.24	9.52±2.33		0.27 (1.1; 0.34; -0.28-0.96)	0.34±2.05	
Anxiety of likelihood of illness						
Intervention	15.27±5.325.32	10.66±5.39	0.41 (0.82; 0.98; -1.37-3.33)	< 0.001 (5.35; 4.67; 2.87-6.33)	4.60±6.10	0.001 (3.43; 3.60; 1.52-5.69)
Control	14.29±6.21	13.29±6.55		0.068 (1.87; 1; -0.07-2.07)	1.00±3.54	
Anxiety of negative consequences						
Intervention	4.09±2.6	2.62±3.21	0.09 (1.67; 0.93; -0.18-2.06)	0.01 (2.55; 1.47; 0.31-2.62)	1.47±4.11	0.12 (1.56; 1.06; -0.28-2.41)
Control	3.15±2.86	2.75±2.76		0.18 (1.36; 0.41; -0.19-1.01)	0.40±1.99	

95%CI = 95% confidence interval.

* p-value for comparison between the control and intervention groups at baseline.

^†^ p-value for comparison between values before and after intervention, within each group separately;

^‡^ p-value for comparison of mean difference (before – after) between the control and intervention groups;

Before the counseling sessions, there were no significant differences in mean scores between two groups for anxiety (p = 0.82) or depression related to coronavirus (p = 0.27), anxiety of likelihood of illness (p = 0.06), or anxiety of negative consequences (p = 0.18). However, after the counseling sessions, mean scores for all the abovementioned variables had significantly decreased in the intervention group (p < 0.01); After the intervention, anxiety related to coronavirus and likelihood of illness were significantly lower in the intervention group than they were in the control group (p = 0.001 and p = 0.001, respectively). Depression related to coronavirus and anxiety of negative consequences decreased non-significantly after the counseling sessions in the intervention group in comparison with the control group (p = 0.08 and p = 0.12, respectively).

## Discussion

For the first stage of the study, in the first months of the COVID-19 pandemic, a web-based cross-sectional survey of Iranian medical staff who work in high-risk situations was conducted with the modified HADS and SHAI.^32^ The results indicated a very high prevalence of anxiety and depression related to coronavirus. The COVID-19 pandemic compromised the psychological health and emotional state of medical staff who have direct contact with infected patients.^33^ This is similar to previous pandemic outbreaks, in that a high incidence of psychopathological responses was detected among medical staff.^13,34-36^ Taking care of infected patients makes them afraid of being infected themselves and of transmitting the disease to their family, friends, and colleagues.^37,38^

Using SHAI to measure cognitive factors associated with hypochondriasis,^39^ this study revealed that the mean values of the total health anxiety score and its two domain scores (the anxiety of likelihood of illness and anxiety of negative consequences) were higher than they were in some other non-clinical samples.^39-42^ It seems this high level of health anxiety was related to the vulnerability of the members of the sample, who worked in close contact with a new emerging and very highly contagious disease with high mortality and morbidity rates. Individuals in different jobs may experience different levels of health anxiety, but those who are at the core of the crisis are affected more.^13,21,43^

Fatal virus pandemics weaken health systems and disrupt plans for protecting the mental health of medical workers and patients.^44^ How to best respond to such challenges during outbreaks is unknown.^45^ Unfortunately, most of the time, the mental health of the staff is ignored in these situations.^9,46^ The lower the level of mindfulness is, the worse staff wellbeing will be.^47,48^

Improving health care staff’s ability to regulate emotions and enhancing effective coping strategies increase the chances of winning the battle against the pandemic.^11,49^ In view of the recommendations on social distancing as well as the crowded and compressed working shifts of medical personnel during the COVID-19 epidemic, attending face-to-face counseling sessions is very inconvenient for medical staff. Therefore, tele-counseling is a better option in this situation^20,33^ and was planned and delivered for the participants in the second stage of this study.

After the counseling sessions, both the level of anxiety related to coronavirus and the level of anxiety related to likelihood of illness were significantly decreased. The psychological intervention delivered in this study was in the line with the recommendations made by Zhang et al. for responding to the COVID-19 epidemic.^31^ The core components of counseling content in this study were cognitive-behavioral and mindfulness-based techniques as well as emotional support that aimed to produce better mental states and coping styles.^50^ Cognitive-behavioral therapy is the most researched and widely recommended treatment for alleviating health anxiety.^51,52^

Overall, there is evidence for the efficacy of specifically designed psychological interventions in conditions of crisis.^51,53^ Even a brief mind-body skill training course was associated with improvements of depression and anxiety.^52^ Mindfulness may serve as a protective factor that alleviates or eliminates the negative effects of perceived stress.^16^ Symptoms of high levels of depression and anxiety of negative consequences were not significantly decreased in this study. This can be attributed to some factors. Firstly, tele-counseling seems to be less effective than face-to-face sessions.^54^ Secondly, the goal of holding intensive sessions in this study was to implement an urgent intervention to control the high incidence of depression and anxiety among staff, to maintain their mental health and clinical performance. However, they did not have enough time to do their homework, which was related to the pressure of high workloads.

Limitations: The use of online systems for data collection and intervention may have caused bias in the randomization. Conducting the study with staff who participated voluntarily may reduce the generalizability of the results.

## Conclusion

It is suggested that hospital managers focus on psychological support for their staff by providing training and counseling services to enhance their coping strategies. Governments should provide psychiatric services for addressing stress and other negative psychological effects of pandemics. Continuous surveillance and monitoring of the psychological status of medical staff both before and during outbreaks should be emphasized.
